# Diagnostic accuracy of plasma NT-proBNP levels for excluding cardiac abnormalities in the very elderly

**DOI:** 10.1186/1471-2318-10-85

**Published:** 2010-11-11

**Authors:** Bert Vaes, Victoria Delgado, Jeroen Bax, Jan Degryse, Rudi GJ Westendorp, Jacobijn Gussekloo

**Affiliations:** 1 Department of General Practice, Université Catholique de Louvain, Avenue Mounier 53, bte 5360, 1200 Brussels, Belgium; 2 Department of Cardiology, Leiden University Medical Center, Leiden, The Netherlands; 3 Department of Gerontology and Geriatrics, Netherlands Consortium for Healthy Aging, Leiden University Medical Center, Leiden, The Netherlands; 4 Department of Public Health and Primary Care, Leiden University Medical Center, Leiden, The Netherlands

## Abstract

**Background:**

In the elderly the diagnosis of chronic heart failure is often challenging and the availability of echocardiography can be limited. Plasma levels of NT-proBNP are valuable tools to diagnose patients with heart failure. However, the performance of this biomarker to detect cardiac abnormalities in the very elderly remains unclear. The aims of this study were to investigate the relation between NT-proBNP and cardiac abnormalities and to evaluate the use of NT-proBNP to exclude structural and functional cardiac abnormalities in a community-based sample of "well-functioning" nonagenarians.

**Methods:**

A diagnostic cross-sectional study embedded within the Leiden 85-plus Study in the municipality of Leiden, the Netherlands. Plasma NT-proBNP levels were measured and 2-dimensional echocardiography was performed in a subgroup of 80 well-functioning nonagenarians. Linear regression analysis was used to explore the relation between NT-proBNP and cardiac abnormalities and ROC curve analysis was used to assess the performance of NT-proBNP to exclude cardiac abnormalities. The upper limit of the lowest tertile of NT-proBNP was used as a cut-off value.

**Results:**

NT-proBNP levels were associated with abnormal left ventricular (LV) dimensions, LV systolic and diastolic function, left atrial enlargement and valvular heart disease. LV mass, E/A ratio and degree of aortic regurgitation were identified as independent predictors of NT-proBNP. NT-proBNP levels were higher with greater number of echocardiographic abnormalities (P < 0.001). A cut-off level of 269.5 pg/mL identified patients with abnormal LV dimensions or depressed LV systolic function (sensitivity 85%, negative predictive value (NPV) 77%, area under the curve 0.75 (95% CI 0.64-0.85)). In addition, high NPV were found for LV systolic dysfunction, left atrial enlargement, severe valvular heart disease and pulmonary hypertension. The test performance of NT-proBNP to exclude any echocardiographic abnormality showed a sensitivity of 82% and a NPV of 65%.

**Conclusions:**

In this convenience sample of well-functioning nonagenarians NT-proBNP was related to a wide variety of functional and structural echocardiographic abnormalities. Moreover, NT-proBNP could be used to exclude echocardiographic abnormalities in well-functioning nonagenarians and might be used to indicate who needs to be referred for further cardiovascular examination.

## Background

Chronic heart failure is becoming more frequent in our aging societies. The prevalence of heart failure increases with age from 0.7% in people aged 55-64 years to 13.0% in those aged 75-84 years [1]. In the elderly, the diagnosis of chronic heart failure is often challenging when there are multiple comorbidities and many other possible causes for dyspnoea, fatigue or peripheral oedema can be present. Echocardiography is currently the diagnostic test of first choice for identifying structural and functional cardiac abnormalities. However, its availability for the very elderly can be limited, so there is the likelihood of a considerable over- and underdiagnosis of heart failure [2,3]. This emphasises the need for a simple test to identify patients at risk.

Plasma levels of BNP (brain natriuretic peptide) and NT-proBNP (N-terminal pro-brain natriuretic peptide) are valuable tools to diagnose patients with heart failure. These measures have shown to provide prognostic information of mortality and major cardiovascular events, not only in patients with chronic heart failure or coronary artery disease but also in the general population, and this can improve patient management [4-8]. In addition, the measurement of these natriuretic peptides has been proposed in screening for left ventricular (LV) dysfunction in high-risk patients, such as the elderly [9,10].

However, multiple factors are known to influence the circulating levels of natriuretic peptides. The prevalence of possible influencing factors, like renal dysfunction and anemia, increases in the very elderly [11,12]. An understanding of these is a prerequisite for the optimal use of these levels as a tool for diagnosing cardiac dysfunction in the community [13]. In the Leiden 85-plus Study a strong and specific correlation between plasma NT-proBNP and various cardiac diagnoses, like atrial fibrillation and myocardial infarction, was found in nonagenarians [14]. However, the performance of this biomarker to detect structural and functional cardiac abnormalities in this subgroup of subjects remains unclear. Therefore, the aims of the present study were first, to study the relation between NT-proBNP levels and structural and functional cardiac abnormalities in a convenience sample of 80 "well-functioning" nonagenarians; second, to evaluate the use of NT-proBNP levels to exclude structural and functional cardiac abnormalities in this age group.

## Methods

### Study Population

The Leiden 85-plus Study is an observational, prospective population-based study of inhabitants of the city of Leiden, The Netherlands. The study design and characteristics of the cohort have been described in detail [15]. In brief, between September 1997 and September 1999 all 705 members of the 1912-1914 birth cohort in the city of Leiden were asked to participate in the month after their 85^th ^birthday. There were no exclusion criteria regarding health status or demographic characteristics. At baseline and yearly up to the age of 90, the participants were visited at their place of residence. For the current study, a convenience sample of 82 participants was invited for echocardiography. The study nurse invited those participants who were physically able to visit the research centre and whose cognitive function allowed them to undergo various technical investigations. All but one underwent echocardiography. All participants in the study gave informed consent and the Medical Ethics Commission of the Leiden University Medical Centre approved the study.

### Functional and Clinical Characteristics

Evaluation of the functional status of participants included measures of cognitive function, subjective well-being, disability and depressive symptoms. Cognitive function was assessed by the Mini-Mental State Examination (MMSE), with scores ranging from 0 to 30 points (optimal) [16]. Subjective well-being was tested with a visual analogue scale (CANTRIL) in those with MMSE >18 points, with scores ranging from 0 to 10 (optimal) [16]. Disability was assessed on the Groningen Activity Restriction Scale (GARS), which is a combination of nine items relating to activities of daily living (ADLs) and nine items relating to instrumental ADLs: scores range from 18 (totally independent) to 72 points (totally dependent) [16]. Depressive symptoms were measured in those with MMSE >18 points, using the 15-item Geriatric Depression Scale (GDS-15), with scores ranging from 0 (optimal) to 15 points [16]. Each participant's general practitioner (or, if applicable, his/her nursing home physician) was interviewed annually about the patient's medical history, using standardised questionnaires including questions on present and past cardiovascular and non-cardiovascular morbidities. Specific cardiac diagnoses were defined as the presence of a medical history of myocardial infarction, angina pectoris, arrhythmias or heart failure, or an electrocardiogram (ECG) at age 90 years revealing a prior myocardial infarction (Minnesota Code 1-1 or 1-2, excluding 1-2-8), atrial fibrillation (Minnesota Code 8-3-1) or LV hypertrophy (Minnesota Code 310, 330 or 340) [17]. Other vascular morbidity was defined as the presence of a diagnosed noncardiac vascular morbidity including stroke and peripheral arterial disease. Non-cardiovascular morbidity was defined as the presence of a medical history of Parkinson's disease, chronic obstructive pulmonary disease, arthroses (including rheumatoid arthritis and polymyalgia rheumatica), malignancies and/or hip fracture.

### Plasma Levels of NT-proBNP at Age 90

Blood samples were taken within the month after every participant's 90th birthday, and were kept frozen at -80°C. In 2006 citrated plasma levels of NT-proBNP were measured in one batch using the NT-proBNP assay of Roche Diagnostics (Mannheim, Germany) on a Roche Modular E-170 automated immunoanalyser. The within-run coefficient of variation was less than 2% and total variation was less than 6% at all levels measured (400-13500 pg/mL).

### Echocardiography

All patients were imaged between January and September 2004, in stable hemodynamic conditions. In the majority of the patients, transthoracic echocardiography was performed the same day of the blood test. In patients who underwent echocardiography at a different day, no cardiac events were recorded between the two tests. The echocardiographic evaluation was performed by one experienced observer blinded to the NT-proBNP levels.

All patients were imaged in the left lateral decubitus position using a commercially available system (Vingmed system/Vivid Seven, General Electric-Vingmed, Milwaukee, WI, USA) equipped with a 3.5-MHz transducer. Echocardiographic images were acquired at a depth of 16 cm in the parasternal and apical views. Left ventricular (LV) dimensions were measured from M-mode images at the parasternal long-axis view. The LV ejection fraction was derived using the Teichholz formula [18]. Left ventricular diameters were indexed to the body surface area. Left ventricular mass was measured according to the formula proposed by Devereux et al. and indexed to the body surface area [19]. Left ventricular hypertrophy was defined by an LV mass index >149 g/m^2 ^in men and >122 g/m^2 ^for women [20].

Left ventricular diastolic function was assessed from pulsed-wave Doppler recordings of the transmitral inflow, measuring the early (E) and atrial (A) wave peak velocities and the E/A ratio [21]. In addition, left atrial dimensions were measured as a morphologic expression of diastolic function [22]. Left atrial anteroposterior diameter was measured from M-mode images at the parasternal long-axis view and left atrial volume was derived according to the cube formula and indexed to body surface area [20].

The function of the mitral, aortic and tricuspid valves were evaluated with color Doppler echocardiography after optimising the gain and Nyquist limit. Standard continuous and pulsed-wave Doppler recordings were acquired. Stenotic and regurgitant valve diseases were evaluated according to semiquantitative and quantitative methods recommended by the American Society of Echocardiography [23]. When tricuspid regurgitation was present, pulmonary artery pressure was estimated using the modified Bernoulli equation [21].

### Confounding Variables

As previously described, plasma levels of NT-proBNP are influenced by gender, body mass index (BMI), renal dysfunction, anemia and medications for heart failure [11,12,24,25]. Therefore, these parameters were defined as possible confounding variables. BMI at age 90 years was assessed by measuring the subject's height and weight. Renal function was estimated using the criteria of the Modification of Diet in Renal Disease Study Group [26]. Anemia was defined as hemoglobin levels <7.5 mmol/L for women and <8.1 mmol/L for men. Data on relevant cardiovascular medications were gathered from each participant's pharmacist, including any use of diuretics, beta-blockers, angiotensin converting enzyme inhibitors, angiotensin II receptor blockers or digoxin.

### Data Analysis

Echocardiographic parameters were compared between tertiles of NT-proBNP using the Jonckheere-Terpstra test or the Chi-squared test with linear-by-linear association.

Levels of NT-proBNP were log-transformed and the association with echocardiographic parameters was explored further using linear regression analysis corrected for confounding variables. Analysis of variance (ANOVA) testing was used to compare the mean LogNT-proBNP values between different categories of increasing number of cardiac abnormalities.

The performance of NT-proBNP to exclude structural and functional abnormalities was assessed using receiver operating characteristic (ROC) curve analysis. The upper limit of the lowest tertile was used as a cut-off value for NT-proBNP and the sensitivity, specificity, negative predictive value (NPV) and positive predictive value (PPV) were calculated. The highest cut-off value with an NPV of 100% was reported. Data analyses were performed using SPSS 16.0 (SPSS Inc., Chicago, IL, USA).

## Results

The initial cohort consisted of 599 participants at age 85 years. A total of 276 individuals survived up to the age of 90 years. Levels of NT-proBNP were determined in 274 participants. Both echocardiographic data and NT-proBNP levels were available in 80 well-functioning subjects. The prevalence of cardiovascular and non-cardiovascular comorbidities was similar for patients with and without echocardiography (Table 1). There was a difference in NT-proBNP levels between both groups (P > 0.05), but the upper limit of the lowest tertile was in the same range (269.5 pg/mL vs 306.7 pg/mL).

**Table 1:** Sociodemographic, Functional and Clinical Characteristics of Participants with and without Echocardiography at Age 90 Years

	Echocardiographic examination		
		
	Yes (*n *= 80)	No (*n *= 194)	
		
	*n *(%)		P*
Sociodemographic characteristics			
Male	26 (33)	50 (26)	0.28
Institutionalized	17 (21)	87 (45)	<0.001
Education ≤ 6 years primary school	38 (48)	132 (68)	0.002
Income ≤ 750€	34 (43)	105 (54)	0.09
Functional characteristics			
Cognitive impairment (MMSE ≤ 18)	0 (0)	72 (38)	<0.001
Poor well-being (CANTRIL < 7)	16 (20)	35 (29)	0.14
Dependent in daily living (GARS ≥ 56)	4 (5)	64 (34)	<0.001
Depression (GDS-15 ≥ 5)	11 (14)	37 (31)	0.003
Clinical characteristics			
Cardiovascular morbidity	48 (60)	132 (68)	0.22
Specific cardiac diagnoses^a^	47 (59)	120 (62)	0.63
Atrial fibrillation on ECG	8 (10.0)	25 (13)	0.49
Diagnosis of heart failure	20 (25)	39 (21)	0.43
Other vascular morbidity^b^	11 (14)	33 (18)	0.45
Cardiovascular risk factors			
Hypertension	47 (59)	104 (55)	0.58
Diabetes mellitus type II	10 (13)	35 (21)	0.12
Non-cardiovascular morbidity^c^	49 (61)	126 (65)	0.56
BMI > 25 kg/m^2^	50 (65)	101 (64)	0.88
GFR < 60 mL/min/1.73 m^2^	43 (54)	100 (52)	0.74
Anemia^d^	11 (15)	51 (28)	0.014
Cardiovascular medication^e^	34 (43)	97 (50)	0.26
NT-proBNP in pg/mL (median, IQR)	389.5 (220.7-779.1)	471.9 (252.8-1655.3)	0.10‡

^a^Including a history of myocardial infarction (clinical or by ECG), angina pectoris, arrhythmias, heart failure and ECG with atrial fibrillation or left ventricular hypertrophy.

^b^Including a history of stroke and peripheral vascular disease.

^c^Including a history of Parkinson's disease, chronic obstructive pulmonary disease, arthritis (including arthrosis, rheumatoid arthritis and polymyalgia rheumatica), malignancies or hip fracture.

^d^Hemoglobin < 7.5 mmol/L for women, <8.1 mmol/L for men.

^e^Including any use of diuretics, beta-blockers, angiotensin converting enzyme inhibitors, angiotensin II receptor blockers or digoxin

* Independent Student's *t*-test; ‡, Mann-Whitney *U *test; IQR, inter quartile range.

Most patients showed normal LV end-diastolic diameter (*n *= 69, 86%) and had a preserved LV ejection fraction (*n *= 73, 91%). According to current criteria, LV hypertrophy was present in 35% (*n *= 9) of men and 45% (*n *= 23) of women (Table 2).

**Table 2:** Echocardiographic Characteristics in Well-Functioning Nonagenarians (*n *= 80)

	Median (IQR)	Normal value *
**LV dimensions and systolic function**		
LV EDD index (mm/m^2^)	28 (24 - 31)	≤33
LV ESD index (mm/m^2^)	17 (14 - 20)	-
Interventricular septal wall thickness (mm)	13 (10 - 14)	≤10
Posterior wall thickness (mm)	11 (10 - 13)	≤10
LV Ejection fraction (%)	67 (60 - 77)	≥50
LV Mass index (g/m^2^)		
Men	132 (105 - 160)	≤149
Women	116 (96 - 145)	≤122
**LV diastolic function**		
E/A ratio	0.7 (0.6 - 0.8)	-
Indexed left atrial volume (ml/m^2^)	24 (14 - 33)	≤40
**Estimated PAP (mmHg)**	34 (30 - 37)	≤40
		
**Valvular function**	**Prevalence, n (%)**	
		
Mitral stenosis	0 (0)	
Mitral regurgitation	58 (72)	
Mild	20 (25)	
Moderate	24 (30)	
Severe	14 (17)	
Aortic stenosis	14 (17)	
Mild (mean ΔP < 25 mmHg)	9 (11)	
Moderate (mean ΔP 25 - 40 mmHg)	4 (5)	
Severe (mean ΔP > 40 mmHg)	1 (1)	
Aortic regurgitation	38 (48)	
Mild	15 (19)	
Moderate	17 (21)	
Severe	6 (8)	
Tricuspid stenosis	0 (0)	
Tricuspid regurgitation	25 (31)	
Mild	8 (10)	
Moderate	4 (5)	
Severe	13 (16)	

Abbreviations: ΔP, pressure gradient; IQR, inter quartile range (25% - 75%); EDD, end-diastolic diameter; ESD, end-systolic diameter; LV, left ventricular; PAP, pulmonary artery pressure.

* References: [20,29].

Patients in the highest NT-proBNP tertile showed significantly larger LV dimensions, higher values of LV mass and a higher E/A ratio together with a larger left atrial volume (Table 3). In addition, a higher prevalence of significant mitral and aortic valve regurgitation was observed in patients in the highest NT-proBNP tertile, together with higher values of pulmonary artery pressure.

**Table 3:** Correlation between NT-proBNP Levels and Echocardiographic Parameters

	Tertiles of NT-proBNP^*^				Adjusted B (95% CI)	
				
	1 (*n *= 26)	2 (*n *= 27)	3 (*n *= 27)	*P *for trend	Model 1**, adjusted R^**2 **^**= 0.19** **Model 2**^**£**^**, adjusted R**^**2 **^**= 0.35**	*P*
**LV dimensions and systolic function**						
LV EDD (mm)	42 (39 - 51)	47 (43 - 54)	51 (45 - 56)	0.003	-0.042 (-0.088 - 0.005)	0.081
LV ESD (mm)	27 (22 - 31)	28 (27 - 33)	32 (29 - 37)	0.002	0.034 (-0.030 - 0.099)	0.29
LV Ejection fraction (%)	69 (60 - 79)	67 (61 - 72)	67 (53 - 74)	0.24	0.006 (-0.017 - 0.030)	0.59
LV Mass index (g/m^2^)						
Men	213 (182 - 287)	236 (177 - 260)	268 (209 - 372)	0.068	0.003 (0.000 - 0.005)	0.045
Women	160 (142 - 190)	212 (169 - 242)	213 (160 - 260)	0.008		
**Left ventricular diastolic function**						
E/A	0.61 (0.51 - 0.70)	0.72 (0.61 - 0.78)	0.70 (0.56 - 1.1)	0.016	0.45 (0.026 - 0.87)	0.038
Left atrial volume (ml)	30.1 (22.1 - 40.0)	43.6 (25.6 - 55.3)	50.3 (33.8 - 73.6)	0.002	0.001 (-0.003 - 0.004)	0.67
**Significant heart valvular disease**						
Mitral regurgitation ≥ moderate	4 (11%)	18 (47%)	16 (42%)	0.003	-0.046 (-0.14 - 0.045)^$^	0.32
Aortic regurgitation ≥ moderate	3 (13%)	8 (35%)	12 (52%)	0.014	0.11 (0.002 - 0.21)^$^	0.046
Any aortic stenosis	7 (50%)	3 (21%)	4 (29%)	0.25	-0.008 (-0.022 - 0.005)^$^	0.23
Estimated PAP (mmHg)	33 (26 - 36)	33 (29 - 37)	37 (33 - 40)	0.015	0.008 (-0.020 - 0.035)^£^	0.56

Abbreviations: EDD, end-diastolic diameter; ESD, end-systolic diameter; LV, left ventricular; PAP, pulmonary artery pressure.

*Echocardiographic parameters (median (IQR)) compared between gender-specific tertiles of NT-proBNP using the Jonckheere-Terpstra test (continuous variables) or the Chi-Square test with linear-by-linear association (proportions). Median values of NT-proBNP levels were 143.2 pg/mL (IQR 103.3-220.7 pg/mL) in the first tertile, 380.6 pg/mL (IQR 326.2-499.4 pg/mL) in the second tertile and 1324.0 pg/mL (IQR 762.4-2832.0 pg/mL) in the third tertile.

** Linear regression analysis with LogNT-proBNP as dependent variable and all echocardiographic measurements (except estimated PAP) as independent variables, adjusted for gender, BMI, renal function, anemia and medications for heart failure (n = 56).

^$ ^In the linear regression analysis the degree of mitral and aortic regurgitation were expressed as ordinal variables (1 to 4) and aortic stenosis was expressed as a continuous variable using the aortic valve peak gradient.

^£ ^Linear regression analysis with LogNT-proBNP as dependent variable and all echocardiographic measurements AND estimated PAP as independent variables, adjusted for gender, BMI, renal function, anemia and medications for heart failure (n = 37).

Linear regression analyses with LogNT-proBNP as the dependent variable and all echocardiographic parameters as independent variables showed that LV mass, E/A ratio and the degree of aortic regurgitation were independent predictors of NT-proBNP after adjusting for confounding variables (Table 3). Two multivariate regression models were used to evaluate the independent determinants of NT-proBNP levels: one including pulmonary artery pressure and another one without.

**Figure 1:**
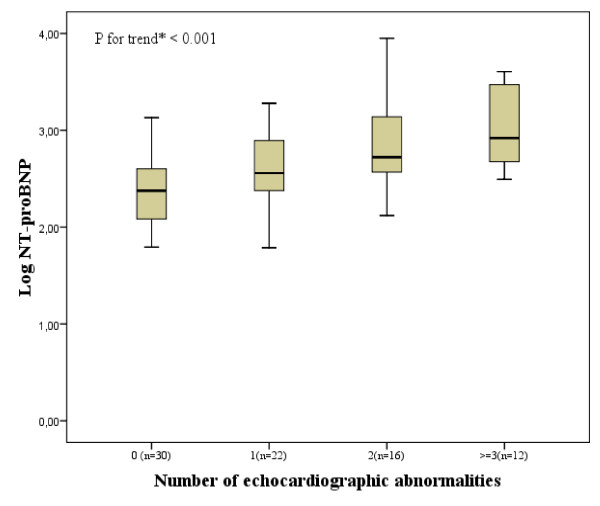
**Relationship between LogNT-proBNP Level and Number of Cardiac Abnormalities at Age 90**. Error bars in these box-and-whisker plots show the mean and 95% confidence intervals of LogNT-proBNP at age 90 related to total amount of cardiac abnormalities, including LVEDD > 33 mm/m^2^, EF < 50%, indexed left atrial volume >40 ml/m^2^, LV hypertrophy (men > 149 g/m^2^, women > 122 g/m^2^), presence of severe mitral regurgitation, presence of severe aortic regurgitation, presence of severe aortic stenosis or PAP ≥ 40 mmHg. * by ANOVA.

The mean LogNT-proBNP was higher with greater number of echocardiographic abnormalities (ANOVA, P < 0.001) (Figure 1). Back transformation yielded the following NT-proBNP levels: 238.2 pg/mL for a normal echocardiographic result, 362.2 pg/mL for patients with one echocardiographic abnormality, 527.2 pg/mL with two and 833.7 pg/mL for patients with three or more.

Because NT-proBNP levels did not differ between men and women (501.1 pg/mL (IQR 211.3-1468.3 pg/mL) and 349.5 pg/mL (IQR 214.9-682.5 pg/mL) respectively (Mann-Whitney, *P *= 0.14)) and gender was no independent predictor of NT-proBNP in the linear regression analysis (*P *= 0.88), no gender-specific cut-off values were used (Table 4). The prespecified NT-proBNP cut-off value of 269.5 pg/mL identified LV dilatation, hypertrophy or systolic dysfunction with sensitivity, specificity, PPV and NPV of 84%, 48%, 59% and 77%, respectively. By reducing the cut-off value to 130 pg/mL, the NPV increased to 100%. The prespecified cut-off value was able to exclude an EF < 50 with an NPV of 92%.

**Table 4:** Test Characteristics for NT-proBNP Levels (Cut-off 269.5 pg/mL) for Detecting Structural and Functional Cardiac Abnormalities in Nonagenarians

	Prevalence *n *(%)	AUC (95% CI)	Sens. (%)	Spec. (%)	PPV (%)	NPV (%)
Abnormal LV dimensions (LVEDD index ≥33 mm/m^2 ^or LV hypertrophy) and/or LV systolic dysfunction	38 (48)	0.75 (0.64-0.85)	84	48	59	77*
LV systolic dysfunction (EF < 50)	7 (9)	0.67 (0.42-0.92)	71	33	9	92*
Indexed left atrial volume ≥ 40 ml/m^2^	12 (15)	0.74 (0.60-0.88)	92	36	21	96^£^
Severe valvular heart disease^#^	21 (26)	0.71 (0.58-0.84)	90	41	35	92^$^
PAP ≥ 40 mmHg	10 (13)	0.85 (0.72-0.98)	100	35	28	100
Severe valvular heart disease and/or LV systolic dysfunction (EF < 50)	25 (31)	0.68 (0.55-0.81)	84	40	39	85^$^
Any severe echocardiographic abnormality	50 (63)	0.77 (0.66-0.88)	82	57	76	65^$^

Area under the curve (AUC), sensitivity, specificity, positive predictive value (PPV) and negative predictive value (NPV) calculated for an NT-proBNP cut-off value of 269.5 pg/mL.

Abbreviations: EDD, end-diastolic diameter; LV, left ventricular; EF: ejection fraction; PAP, pulmonary artery pressure; CI, confidence intervals.

An NT-proBNP cut-off value of 130 pg/mL (*), 236 pg/mL (^£^) and 61 pg/mL (^$^) increased the NPV to 100%.

^# ^Severe mitral or aortic stenosis, severe mitral regurgitation and severe aortic regurgitation.

To identify left atrial enlargement, severe valvular heart disease and pulmonary hypertension, the prespecified cut-off value yielded a high sensitivity and NPV but low specificity and PPV. An NT-proBNP cut-off value of 61 pg/mL excluded the presence of severe valvular heart disease.

The test performance of NT-proBNP to exclude severe valvular heart disease and/or LV systolic dysfunction showed a sensitivity of 84% and a NPV of 85%. In addition, the diagnostic accuracy of NT-proBNP to exclude any echocardiographic abnormality (n = 50) was calculated. If only the participants with an NT-proBNP level >269.5 pg/mL (n = 54) would be referred for echocardiography, 9 cases would be missed (18%) and 13 negative echoes (0 abnormalities) would be done (43%).

## Discussion

In this convenience sample of well-functioning nonagenarians we showed NT-proBNP is not just an indicator of increased cardiac pressure but is related to a wide variety of functional and structural echocardiographic abnormalities. This confirms the hypothesis that natriuretic peptides could possibly be used to identify 'pancardiac' damage, even when it is silent [27]. In a previous study in this cohort NT-proBNP was found to be a good predictor for both cardiovascular and noncardiovascular mortality, both in elderly with and without specific cardiac diagnoses. This indicated elevated plasma NT-proBNP levels possibly reflect unknown cardiac morbidity or imminent heart failure [14]. In this convenience sample 18 of 33 patients (56%) without specific cardiac diagnoses had one or more echocardiographic abnormality. Moreover, increasing levels of NT-proBNP were related to the number of echocardiographic abnormalities.

A recent systematic review concluded important questions about the implementation of measuring NT-proBNP in daily practice remain unsolved for the diagnosis of cardiac dysfunction in elderly patients aged 75 and over from the general population [28]. The present study confirms that low NT-proBNP levels are most efficient in excluding echocardiographic abnormalities in well-functioning nonagenarians. Possibly the level of NT-proBNP might be used to indicate who needs to be referred for further cardiovascular examination including echocardiography.

Some limitations should be acknowledged. First, we were not able to correlate levels of NT-proBNP to echocardiographic measurements for the entire cohort. Seen the small sample of well-functioning nonagenarians we should be careful to generalize the findings of the present study to the entire population of well-functioning nonagenarians. Second, there was no available data on the symptomatology of the participants. However, participants in the convenience sample only differed in functional status, but had the same burden of cardio-vascular comorbidities compared to the limited-functioning elderly. Third, the fact the NT-proBNP levels were determined in 2006 after being frozen might have influenced the absolute value, but it is unlikely that it has affected the ranking. In addition, the applicability of current echocardiographic cut-off values to nonagenarian subpopulations may be debatable since the thresholds for normal values are usually derived from younger subpopulations. Finally, LVEF measured with biplane Simpson's methods was not systematically available in all patients.

## Conclusions

In conclusion, this study showed NT-proBNP is related to a wide variety of functional and structural echocardiographic abnormalities in well-functioning nonagenarians. Furthermore, low values of NT-proBNP were able to exclude echocardiographic abnormalities and could be used to identify nonagenarians that need to be referred for further cardiovascular examination.

## List of abbreviations

BNP: Brain natriuretic peptide; NT-proBNP: N-terminal pro-brain natriuretic peptide; ROC: Receiver operating characteristic; LV: Left ventricular; CI: Confidence intervals; MMSE: Mini-Mental State Examination; GARS: Groningen Activity Restriction Scale; ADLs: Activities of daily living; GDS-15: 15-item Geriatric Depression Scale; BMI: Body mass index; ANOVA: Analysis of variance; NPV: Negative predictive value; PPV: Positive predictive value; ΔP: Pressure gradient; SD: Standard deviation; IQR: Inter quartile range; EDD: End-diastolic diameter; ESD: End-systolic diameter; PAP, Pulmonary artery pressure.

## Competing interests

JB has received grants from Biotronik (Berlin, Germany), Medtronic (Minneapolis, MN, USA), Boston Scientific Corporation (Natick, MA, USA), Bristol-Myers Squibb Medical Imaging (New York, NY, USA), St. Jude Medical (Saint Paul, MN, USA), GE Healthcare (Milwaukee, WI) and Edwards Lifesciences (Irvine, CA, USA).

## Authors' contributions

JG had full access to all of the data in the study and takes responsibility for the integrity of the data and the accuracy of the data analysis. JG and RGJW are responsible for the study concept and design. BV and VD performed the statistical analysis and drafted the manuscript. All authors participated in the analysis and interpretation of the data and critically revised the manuscript.

## Pre-publication history

The pre-publication history for this paper can be accessed here:

http://www.biomedcentral.com/1471-2318/10/85/prepub
